# Metastable Pain-Attention Dynamics during Incremental Exhaustive Exercise

**DOI:** 10.3389/fpsyg.2016.02054

**Published:** 2017-01-06

**Authors:** Agnė Slapšinskaitė, Robert Hristovski, Selen Razon, Natàlia Balagué, Gershon Tenenbaum

**Affiliations:** ^1^Complex Systems in Sport Research Group, INEFC Barcelona UniversityBarcelona, Spain; ^2^Saints Cyril and Methodius University of SkopjeSkopje, Macedonia; ^3^West Chester UniversityWest Chester, PA, USA; ^4^Florida State UniversityTallahassee, FL, USA

**Keywords:** non-linear dynamics, pain, attention, accumulated effort, exercise, metastability

## Abstract

**Background:** Pain attracts attention on the bodily regions. Attentional allocation toward pain results from the neural communication across the brain-wide network “connectome” *which consists of pain-attention related circuits*. Connectome is intrinsically dynamic and spontaneously fluctuating on multiple time-scales. The present study delineates the pain-attention dynamics during incremental cycling performed until volitional exhaustion and investigates the potential presence of nested metastable dynamics.

**Method:** Fifteen young and physically active adults completed a progressive incremental cycling test and reported their discomfort and pain on a body map every 15 s.

**Results:** The analyses revealed that the number of body locations with perceived pain and discomfort increased throughout five temporal windows reaching an average of 4.26 ± 0.59 locations per participant. A total of 37 different locations were reported and marked as painful for all participants throughout the cycling task. Significant differences in entropy were observed between all temporal windows except the fourth and fifth windows. Transient dynamics of bodily locations with perceived discomfort and pain were spanned by three principal components. The metastable dynamics of the body pain locations groupings over time were discerned by three time scales: (1) the time scale of shifts (15 s); (2) the time scale of metastable configurations (100 s), and (3) the observational time scale (1000 s).

**Conclusion:** The results of this study indicate that body locations perceived as painful increase throughout the incremental cycling task following a switching metastable and nested dynamics. These findings support the view that human brain is intrinsically organized into active, mutually interacting complex and nested functional networks, and that subjective experiences inherent in pain perception depict identical dynamical principles to the neural tissue in the brain.

## Introduction

Humans have the capacity to distract from and also differentiate between various sensations related to physical exercise such as exercise-related effort and pain (O’Connor and Cook, 2001; Pageaux, 2016). Perception of effort is defined as “the conscious sensation of how hard, heavy, and strenuous a physical task is" (Marcora, 2010). Perception of pain on the other hand is defined as the perception of a distressing experience associated with actual or potential tissue damage that entails sensory, cognitive, emotional, and social components (Williams and Craig, 2016). From a standpoint of measurement accuracy within self-report settings, the instructions provided by the test administrator play an important role for distinguishing between perception of effort and pain (Pageaux, 2016).

Of specific interest herein, perception of pain typically requires attentional allocation. To that end, attention focus is a key cognitive mechanism for increasing or decreasing the perception of pain (Legrain et al., 2009; Slapsinskaite et al., 2015). Even in the presence of unchanging nociceptive input and regardless of on-going task demands the attentional state seems waxing and waning spontaneously (Peters and Crombez, 2007). Consequently, shifting of attention could be due to the pain-attention related processes that are intrinsically dynamic and spontaneously fluctuating on multiple time-scales (Bressler and Kelso, 2001, 2016; Deco et al., 2013). Pervasiveness of such attentional fluctuations, their intrinsic nature, and their relevance to subjective experience, such as pain, are further supported by the evidence provided from studies of spontaneous brain dynamics and the impact of pre-existing brain state on subsequent perception (Kucyi and Davis, 2015). Within such a framework, attentional fluctuations away from non-painful modalities and their neural mechanisms can be termed “perception decoupling” or “disengagement of attention” from perception (Schooler et al., 2011). Spontaneous attentional fluctuations toward and “away from pain” and individual differences in this regard are represented in the very brain network structure and dynamics.

The dynamical system theory (DST) is a sub field of mathematics that aims at understanding and describing the dynamical changes that occur over time. Specifically, DST establishes a series of principles that govern the system’s dynamical changes. In the last few decades, DST has demonstrated that painful experiences are emergent phenomena resulting from self-organized processes, and that pain-attention interaction can be understood as a virtue of such dynamics (Lutz et al., 2008). To that end, it is known that non-linear dynamic mechanisms are involved in the modulation of attentional focus during physical activity (Balagué et al., 2012; Slapsinskaite et al., 2016). Indeed, the DST framework that captures pain in terms of spatiotemporal trajectories of neural activity emerging from complex non-linear neural interactions, provides a novel approach to the study of pain-attention dynamics (Freeman, 1992).

With regards to the perception of painful sensations also known as nociception (Pfaff, 2013), the brain is intrinsically organized into active, mutually interacting complex and functional networks. There is also a consensus that the pain experience is both highly subjective and top-down modulated (Garland, 2013). To that end, evidence indicates that non-linear dynamical processes form the basis of a number of neural (Izhikevich, 2010) and higher order processes. Specifically, non-linear processes are defined as those with non-proportionality between the input and the output and with occasional reduction to linear processes (Kelso, 1997).

Findings from research that focused on subjective experiences have revealed fluctuating and metastable dynamics inherent to effort perception and within different types of exercise setting (Balagué et al., 2012, 2015; Aragonés et al., 2013; Garcia et al., 2015; Slapsinskaite et al., 2016). Metastability can be seen as a property related to the existence of multiple separated timescales (Bovier and Den Hollander, 2016). At short time-scales, the system appears to be in equilibrium, but in fact, explores only a limited part of its available state space. At longer timescales, it undergoes transitions between numbers of metastable states.

From a broader standpoint, overall cognition is also facilitated through the dynamical phenomenon of chunking (i.e., larger sequence of information is managed into its smaller units). Indeed, chunking has been shown to be involved in a range of perception and cognition-related processes in humans (Gobet et al., 2001; Rabinovich et al., 2014). Thus, the notion that mental function is based on the dynamical and ongoing interaction of a number of neural and bodily parts that produce complex patterns has gained acceptance (Thompson and Varela, 2001; Rabinovich et al., 2008; Rabinovich and Muezzinoglu, 2010).

To date, it is known that brain exhibit periods of stability and instability in both behavioral and neural levels. The transition from stable to unstable patterns comes as a response to the changes in control parameters as those govern the system’s properties (stability and instability) (Haken, 1987). The neural dynamics of the brain, based upon metastability and dwelling on different time scales, flexibly reorganize pain-attention on a moment to moment basis. Consequently, the processes that are more stable dwell over longer time scales and naturally tend to correlate with the pain-attention configurations that emerge over shorter time scales. These dynamics, in turn, are reflected in the sequential switching in between the temporally and structurally nested metastable states during trials (Rabinovich and Varona, 2011).

Of specific interest herein, the link among the dynamic principles of spontaneous attention fluctuations, brain networks dynamics and the neural processing of pain observed through subjective experiences of pain may be essential for the understanding of attentional modulation and its involvement in the perception of pain. To that end, exercise settings can provide a particularly adequate context because during exercise the sensory, cognitive, emotional, and physical conditions change continuously. On a practical note, capturing pain-attention interaction dynamics through subjective experiences can also contribute to designing non-invasive approaches to ultimately control pain during exercise or beyond. The purpose of this study was to delineate the pain-attention dynamics during incremental cycling performed until volitional exhaustion. Specifically, drawing upon a DST approach, we hypothesized that pain-attention relationship during exercise would display hierarchically or nested metastable dynamics.

## Materials and Methods

### Study Design and Participants

Fifteen young and physically active adults (10 women, 5 men, *M*age = 22.5 years, *SEM* = 0.43, age range: 20–25 years, and *BMI* = 22.84, *SEM* = ± 0.77) who engaged in a wide range of aerobic activities (e.g., jogging, swimming, dancing) at least three times a week, participated in this study. None of the participants had previous history of chronic pain or musculoskeletal injuries at the time of the study. Prior to the onset of the study, participants completed a health history questionnaire, as well as an informed consent form, which was approved by the Clinical Research Ethics Committee of the Sports Administration of Catalonia (registration number 072015CEICEGC). This study was carried out in accordance with the Declaration of Helsinki.

### Discomfort and Pain Monitoring

**FIGURE 1:**
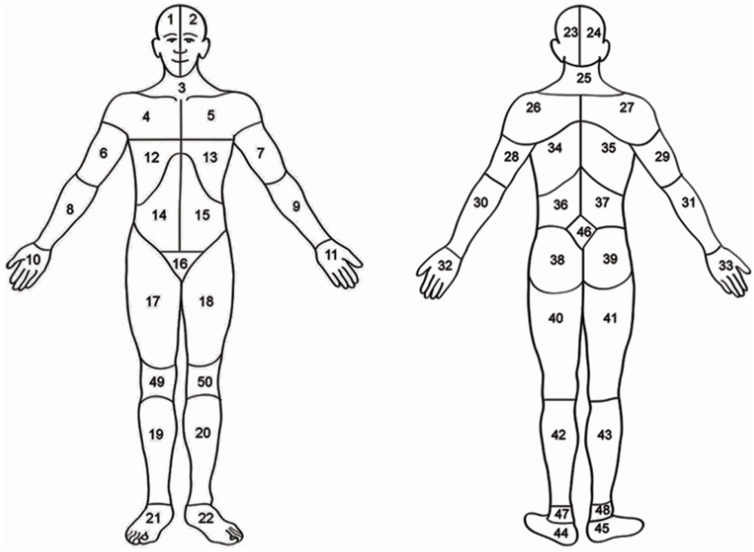
**Body map**. Head (areas 1, 2, 23, or 24); neck (areas 3 or 25), shoulders (areas 4, 5, 26, 27); arms (areas 6, 7, 8, 9, 28, 29, 30, or 31); hand (areas 10, 11, 32, 33); ribs or chest (areas 12 or 13); abdomen (areas 14 or 15), back (areas 34, 35, 36, 37), buttocks or hips (areas 38 or 39); genitalia (area 16), legs (areas 17, 18, 19, 20, 40, 41, 42, or 43); feet (areas 21, 22, 44, or 45). Adapted from Margolis et al. (1986).

To detect pain dynamics and corresponding bodily regions, a body map (see **Figure 1**) was verbally explained to participants prior to the baseline test and experimental tasks. Using the map, every 15 s during exercise, upon the researcher’s prompts, participants reported bodily regions with discomfort and pain. The instructions provided to the participants included the following:

“When prompted, we ask you to report the locations of discomfort and pain (if you feel it, independently of its magnitude) using the numbers on the body map placed in front of you.”

### Familiarization Procedures

All participants were already familiar with cycle ergometer testing. One week prior to the tests, they received instructions on how to use the body map during the tests. To ensure their competence, they practiced a submaximal version of the incremental cycling test (see below) and using the body map, reported bodily regions with pain every 15 s upon the researcher’s prompts. All participants displayed adequate competence of the study protocols following one single trial.

### Incremental Cycling Test

Following a 2 min rest period, participants performed a progressive incremental test on a cycle ergometer (Sport Excalibur 925900) with saddle and handlebar specifications adjusted to their preference. For the purposes of the test, they were instructed to pedal at 60 rpm with an initial load of 30 W and increases of 25 W/min for female and 30 W/min for male, until they could no longer maintain the pedaling rate for five consecutive seconds while in the sitting position. Participants performed the test with no verbal communication except for indicating bodily locations with pain after the researcher’s prompts. Heart rate was continuously monitored (Polar RS 400) to assure that participants reached at least 170 beats/min at the point of exhaustion. Upon task completion, using an 11-point Likert-type scale with anchors ranging from 0 (*not at all*) to 10 (*greatly*), participants answered two questions to measure task commitment: (a) “*Have you pedaled as long as you can, achieving your exhaustion point?*” and, commitment to the protocol (b) “*Have you reported all the changes in your discomfort and pain-locations when required?”*

### Statistical Analysis

The reported number of locations with discomfort and pain during the test were plotted for each participant. Each time series was divided into five non-overlapping temporal windows (time to volitional exhaustion of the participant/5). Mean value of the number of locations with discomfort and pain in total were computed for each time window. The changes of entropy were computed within five temporal windows. A median was calculated for each window from all participants’ mean value of the number of locations with discomfort and pain frequencies of painful bodily locations.

These collected data with painful locations obtained from the body maps were then used to form 17 Boolean *m* × *n* data matrices where *m* signifies the number of body locations and *n* the number of time samples (Casari et al., 1995; Gogos et al., 2000; Jolliffe, 2014). Visual observation of the structure of the data matrices helped distinguish between two types of reported locations: locations that were persistent throughout the entire exercise bout (i.e., long-term and stable locations), and locations that were inconsistent (i.e., short-term and unstable locations). In other words, the data matrix either depicted long-term bodily locations that were stable on the time scale of 10s of minutes, or other short-term ones that were stable on the time scale of 10s of seconds or minutes.

Collective variables were then determined by means of a principal component analysis (PCA) (Jirsa et al., 1994). The Kaiser-Guttmann criterion (eigenvalue λ ≥ 1) was used to define the number of salient PCs of the first order (Yeomans and Golder, 1982). The hierarchical analysis of oblique principal components (hPCA) (Fabrigar et al., 1999) was subsequently used to check for the presence of collective variables of higher order, and to obtain a maximal dimensional reduction of the data. For the purposes of the hPCA analysis, the software package Statistica 5.0 was used.

The null hypothesis of a constant median (with no significant differences) over time was tested using non-parametric repeated-measures Friedman ANOVA. Effect sizes (Cohen’s *d*) were computed to demonstrate the magnitude of standardized differences in medians where effect sizes neared *p* < 0.05 level.

## Results

**FIGURE 2:**
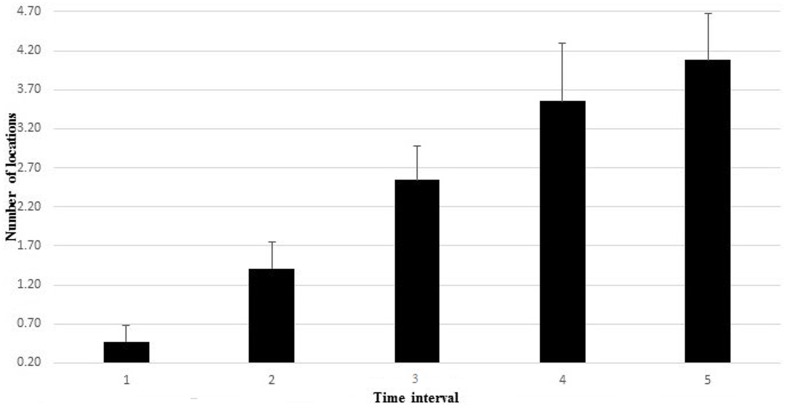
**The number of locations with discomfort and pain throughout the five temporal windows**.

During incremental cycling, the reached maximal load corresponded to 228 ± 17 and 240 ± 30 W for females and males, respectively. On average, participants’ heart rate reached 180 ± 9.5 bpm at the exhaustion point. The Friedman ANOVA revealed a significant effect of time for the total number of locations with discomfort and pain, χ^2^(15,4) = 49.249, *p* < 0.001, during the incremental cycling test. **Figure 2** depicts the changes in the number of locations with discomfort and pain throughout the five temporal windows. The number of locations resulted in a significant difference between temporal windows: first vs. third time intervals, *Z* = -2.97; *p* < 0.05, *d* = 1.59, 95% CI [0.65, 2.04]); third vs. fifth time intervals, *Z* = -3.26; *p* < 0.05, *d* = 0.81, 95% CI [-0.35, 1.73]), and first vs. fifth time intervals, *Z* = -2.17; *p* < 0.05, *d* = 2.27, 95% CI [1.11, 2.74]).

**FIGURE 3:**
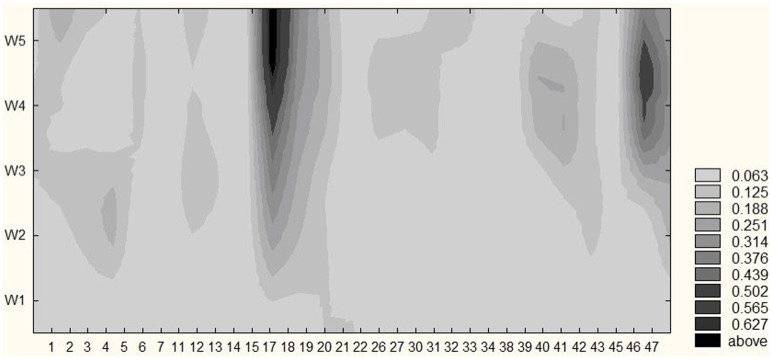
**Locations with pain and/or discomfort**. The group pulled probabilities of locations with pain or discomfort during cycling tasks in five temporal windows in a given sample (*n* = 15). As time on task increases (vertical axis) the number of locations and the probability of experiencing pain and discomfort at selective locations also increase (darker shades of gray) on average. Legend: the probability of experiencing discomfort and pain.

**Figure 3** illustrates the frequencies of locations with discomfort and pain during the incremental cycling test. The number of locations and the probability of experiencing discomfort and pain at select locations (depicted in darker shades of gray), increased during the test until reaching 4.26 ± 0.59 in the fifth temporal window. The dominant locations with discomfort and pain at exhaustion included left and right quadriceps, lower back and left ankle. Both the waxing and waning experience of pain were also identified. Depicted in shades of gray in **Figure 3**, exertive pain exhibited metastable dynamics, dwelling around select bodily regions for some time to transition into another one quickly after. A total of 37 different areas were reported and marked as painful for all participants throughout the cycling test.

**FIGURE 4:**
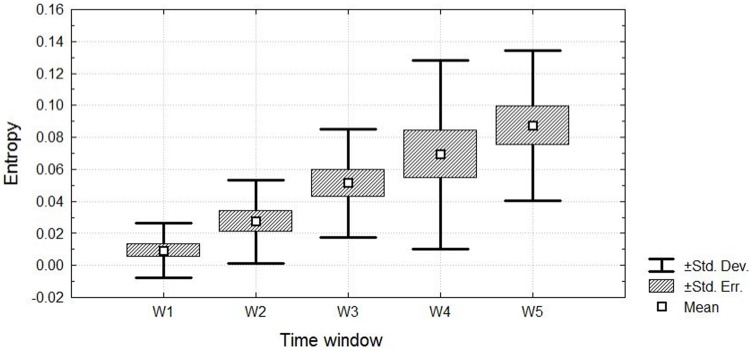
**The entropy of exertive pain throughout the five temporal windows**.

The Friedman ANOVA revealed a significant effect of time for the pain entropy, χ^2^(15,4) = 49.77, *p* < 0.001 in incremental cycling (see **Figure 4**). The entropy of exertive pain showed significant difference between all temporal windows except the fourth and fifth windows. first vs. second time intervals, *Z* = 2.93; *p* < 0.05, *d* = 1.04, 95% CI [1.02, 1.05]; first vs. third time intervals, *Z* = 3.29; *p* < 0.001, *d* = 2.07, 95% CI [2.06, 2.08]; first vs. fourth time intervals, *Z* = 3.4; *p* < 0.001, *d* = 1.62, 95% CI [1.61, 1.63); first vs. fifth time intervals, *Z* = 3.4; *p* < 0.001, *d* = 2.44, 95% CI [2.42, 2.45]; second vs. third time intervals, *Z* = 3.17; *p* < 0.001, *d* = 1.04, 95% CI [1.02, 1.05]; second vs. fourth time intervals, *Z* = 3.17; *p* < 0.001, *d* = 0.81, 95% CI [0.8, 0.82]; second vs. fifth time intervals, *Z* = 3.26; *p* < 0.001, *d* = 1.62, 95% CI [1.61, 1.63]; third vs. fourth time intervals, *Z* = 2.07; *p* < 0.05, *d* = 0.00, 95% CI [-0.01, 0.02]; and third vs. fifth time intervals, *Z* = 3.26; *p* < 0.05, *d* = 0.81, 95% CI [0.8, 0.82]. Kendall’s W was equal to 0.83 with an average rank *r* = 0.82. In general, relative to participants who started with low entropy, participants with higher entropy kept and ended with higher entropy.

**FIGURE 5:**
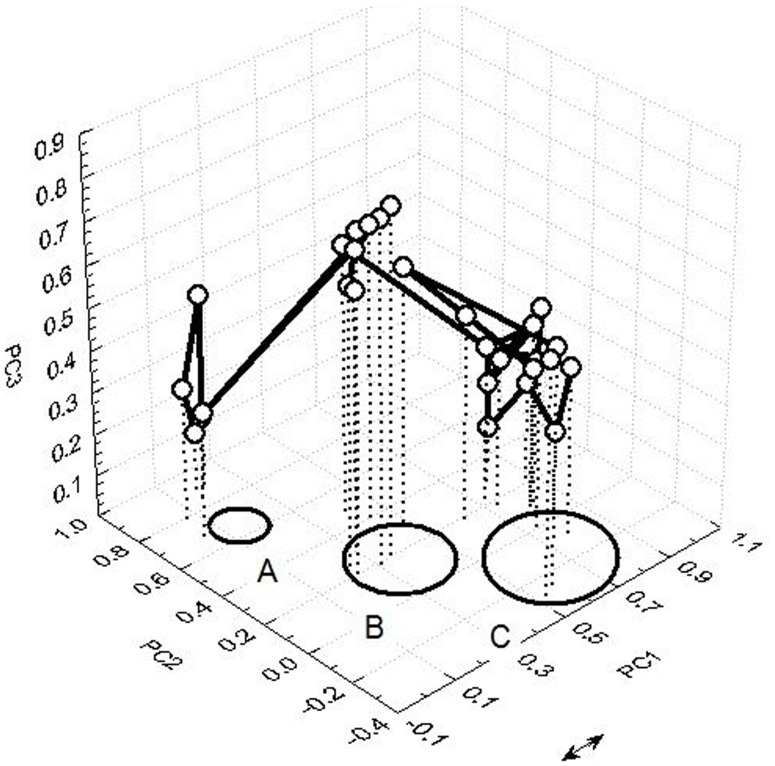
**Transient dynamics of bodily locations with perceived pain spanned by three PCs**.

**Figure 5** depicts an example of transient dynamics of bodily locations with perceived discomfort and pain in the space spanned by three PCs. From a chunk sequence – trajectory within three PCs a dwelling time around select region (e.g., PC1) and a transitional trajectory to another state “temporal winner” PC2 and a final rapid switch to the next metastable state can be identified. This example illustrates a metastability of sensory interactions and integration of information from the painful locations.

**FIGURE 6:**
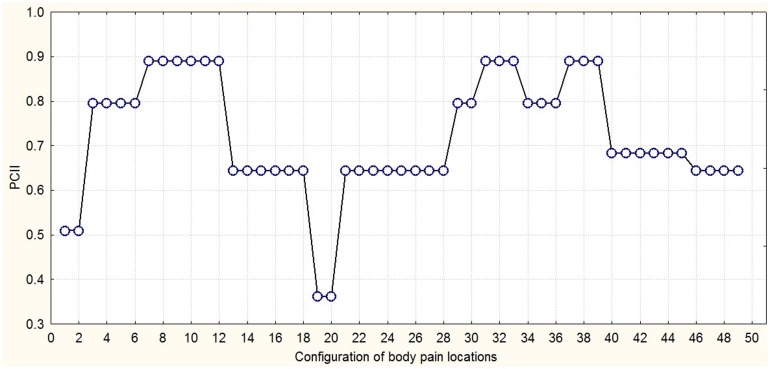
**The metastable dynamics of the configuration of body pain locations over time projected on the secondary PC**.

**Figure 6** presents an integration-segregation picture of exertive pain through three PCs joined hierarchically into one second-order PC. Through hierarchical PC of time series of exertive pain configurations chunks on different time scales can be detected. The trajectory dwells for some time in space then wanes to finally dwell again. The persistent (longest dwell time) painful locations over all time resulted in the emergence of super-chunks. These group-clusters of persistent locations formed a skeleton-like figure on which other less persistent (shorter dwell time) motives join and dissolve following which further short-lived (shortens dwell time) painful locations emerged. The metastable dynamics of the body pain locations groupings over time projected on the secondary PC. The system dwells for some time in one configuration state then quickly shifts to another one. At least three time scales can be discerned: (1) the time scale of shifts (15 s), (2) the time scale of metastable configurations (100 s), and (3) the observational time scale (1000 s).

## Discussion

The present findings advance the understanding of generated sequential switching nature of attention-pain dynamics, and further support the view that human brain is intrinsically organized into active, mutually interacting complex and nested functional networks (Rabinovich and Varona, 2011). Surprisingly, our results draw attention to a potential link between pain-attention dynamic states and modeled sequences of two psychological components of effort, namely cognition and emotion (Rabinovich et al., 2010, 2015), as well as the dynamical signatures of several brain functions and mental diseases (Rabinovich and Varona, 2011). This is an important finding in that it demonstrates that the phenomenological subjective experiences, so called *qualia* (Chalmers, 1996), are grounded in identical dynamical principles to the neural tissue, i.e., the brain.

Consequently, perception of pain and attention to pain seem to be multidimensional and interrelated through hierarchical dynamical processes that highly depend on sensory cues (i.e., painful locations) (Rabinovich et al., 2015). In the light of these findings, it is important to note that attention does not appear to be a static capacity but rather a process that involves the attentional “reorienting” from one input (i.e., one painful location) or modality (i.e., pain intensity or quality) to another. Therefore, the perception process requires a short-term integration between a number of continuously interplaying components such as the environmental cues, the body and the brain itself (Rabinovich et al., 2015).

The present study focused on the evolving interactions among pain-attention dynamics, where perceived pain throughout a cycling task was considered a bi-product of the mutual interaction between attention and distinct psychophysiological process, and not of select singular mechanisms. In fact, disengagement of attention from perception within ongoing dynamics of pain-attention or so called *perceptual decoupling* could also be responsible for the metastable dynamics observed in this study. Studies on neural mechanisms of spontaneous attentional fluctuations on multiple timescales and pain variability have already underlined the importance of dynamics of pain-attention interactions, and its mutual influence on each other. Upcoming work must consider this phenomenon (Kucyi and Davis, 2015).

The present findings also help detect and confirm the dynamical phenomenon of chunking that the biological-cognitive system uses to manage larger sequence of information into smaller units to facilitate information processing. Indeed, the presence of interconnected pieces that are prevalent over long periods of time supports the notion of a hierarchical organization of neural processing, which is the basis for understanding chunking dynamics (Rabinovich et al., 2014). Specifically, in the present study we observed the produced hierarchical chunking of locations with pain sequences, and to our knowledge for the first time, demonstrated how dynamics of mental hierarchies may be established on component perceptions that dwell over different time scales. Our data suggest that basic functions, such as focusing on the painful locations, and chunking of the information evolve through dynamic and not static interactions. That is, while forming a chunking network individuals tend to transform the chain of metastable states along with transient process to the chain of groups of such states. Therefore, within the present framework, it was considered that the chunks operate on an heteroclinic cycle of metastable states where each metastable state itself is a heteroclinic cycle of basic information items (Rabinovich et al., 2014). Altogether, the set of informational items (i.e., painful locations) can be interpreted as sequences. Consequently, conceptualizing pain and attention-related brain and body network processes from the standpoint of a concurrent activation of sensory cues emanating from the body and multiple other sources within a distributed brain network can prove beneficial.

Several limitations to our study should be noted. First, we have not studied all the spatiotemporal mental fields (e.g., alternative facets of perception, cognition, emotion, mental resources) and their dynamics within the exercise setting. Second, magnetic resonance imaging was not used to capture the neurophysiological mechanisms behind the interaction of attention-pain and this may have shed more light on the pain dynamics. Third, pre-existing brain state was not measured and finally, participants’ personal beliefs or expectations about pain were not evaluated. Finally, prompting protocol used herein may also present a limitation. It is plausible that, due to the reporting task, participants’ attention focus was somewhat biased. This protocol was implemented, however, to follow a systematic and regularly imposed rating strategy. Traditionally, the data of pain ratings were obtained in lower recording frequency, for instance varying between 1 and 3 min intervals (Angius et al., 2015), before and after physical activity (Choi et al., 2013), or once per day (Burnett et al., 2010). Some researchers have also recorded pain ratings at high frequencies with use of 30 s (Cook et al., 1998) or 15 s (Slapsinskaite et al., 2015). Intra-individual changes are, however, better captured through short frequency recording of self-reports precisely because participants may not be able to attend and report all changes within lower recording frequency settings. To that end, with regards to the present study, it is important to note that the test administrator was frequently prompting the self-report with no prompting of any particular pain location *per se*.

## Conclusion

To the best of our knowledge, this study remains a first attempt to illustrate and explain the pain-attention information processing dynamics within an exercise setting. Finally, from a translational standpoint greater knowledge into pain dynamics during exercise can help practitioners design effective strategies to cope with painful sensations during effort. This is important in that within effortful settings, somatic pain is associated with negative affective responses to exercise and eventual lack of exercise-adherence (Ekkekakis et al., 2011). Consequently, strategies to allow improved pain management during activity are likely to facilitate exercise-related enjoyment, and help long term exercise-engagement (Saanijoki et al., 2015).

## Author Contributions

Conceived and designed the experiments: NB, RH, AS; performed the experiments: AS, NB; analyzed the data: AS, NB, RH, GT, SR; contributed reagents/materials/analysis tools: RH, AS, NB, SR, GT; wrote the paper: AS, SR, NB, RH.

## Conflict of Interest Statement

The authors declare that the research was conducted in the absence of any commercial or financial relationships that could be construed as a potential conflict of interest.
